# Financial risk of road traffic trauma care in public and private hospitals in Addis Ababa, Ethiopia: A cross-sectional observational study

**DOI:** 10.1016/j.injury.2021.11.009

**Published:** 2022-01

**Authors:** Hailu Tamiru Dhufera, Abdulrahman Jbaily, Stéphane Verguet, Mieraf Taddesse Tolla, Kjell Arne Johansson, Solomon Tessema Memirie

**Affiliations:** a Department of Global Public Health and Primary Care, University of Bergen, Norway; b Department of Global Health and Population, Harvard T.H. Chan School of Public Health, Boston, MA, United States; c Department of Pediatrics and Child Health, College of Health Sciences, Addis Ababa University, Addis Ababa, Ethiopia

**Keywords:** Out-of-pocket expenditure, Catastrophic health expenditure, Trauma care cost, Road traffic injury, Priority setting

## Abstract

**Background:**

Road traffic injuries are among the most important causes of morbidity and mortality and cause substantial economic loss to households in Ethiopia. This study estimates the financial risks of seeking trauma care due to road traffic injuries in Addis Ababa, Ethiopia.

**Methods:**

This is a cross-sectional survey on out-of-pocket (OOP) expenditures related to trauma care in three public and one private hospital in Addis Ababa from December 2018 to February 2019. Direct medical and non-medical costs (2018 USD) were collected from 452 trauma cases. Catastrophic health expenditures were defined as OOP health expenditures of 10% or more of total household expenditures. Additionally, we investigated the impoverishment effect of OOP expenditures using the international poverty line of $1.90 per day per person (adjusted for purchasing power parity).

**Results:**

Trauma care seeking after road traffic injuries generate catastrophic health expenditures for 67% of households and push 24% of households below the international poverty line. On average, the medical OOP expenditures per patient seeking care were $256 for outpatient visits and $690 for inpatient visits per road traffic injury. Patients paid more for trauma care in private hospitals, and OOP expenditures were six times higher in private than in public hospitals. Transport to facilities and caregiver costs were the two major cost drivers, amounting to $96 and $68 per patient, respectively.

**Conclusion:**

Seeking trauma care after a road traffic injury poses a substantial financial threat to Ethiopian households due to lack of strong financial risk protection mechanisms. Ethiopia's government should enact multisectoral interventions for increasing the prevention of road traffic injuries and implement universal public finance of trauma care.

## Introduction

Financial risk protection is a primary objective of health systems [1,2]. Countries now aim to achieve universal health coverage (UHC), defined as all people having access to needed health services without financial hardship [3]. However, economic hardship from out-of-pocket (OOP) health expenditures is substantial [4,5]. Globally, about 800 million people are at risk of catastrophic health expenditures (CHE), and about 100 million individuals are at risk of impoverishing health expenditures (IHE) each year due to seeking health care [4,5].

The high level of OOP expenditures on health care is an important cause of poverty, especially in countries where OOP expenditures are the primary financing source for health systems [4,5]. Road traffic injury (RTI) related emergency care can be a significant contributor to OOP expenditures, with CHE and IHE due to severe injuries and expensive lengthy hospital stays in many low- and middle-income countries (LMICs) [6], [7], [8], [9].

The burden of injury has increased rapidly in LMICs over recent years [10]. In particular, RTIs were the leading cause of injury globally and accounted for 26% of the injury burden in 2017 [10]. RTIs disproportionally affect LMICs, where 85% of all global RTI fatalities were reported in 2018 by the World Health Organization [11]. With 27 deaths per 100,000 population, Ethiopia has one of the highest RTI death rates in sub-Saharan Africa [10], [11], [12], [13].

The broader cost of health care can be composed of direct medical costs (such as the costs of medication, imaging, and laboratory, physician consultation fees, hospital stays, procedures) and direct non-medical costs (such as transportation costs, attendant expenses, and food expenses).

Around 30% of all health care costs for road traffic injuries come from direct OOP payments in Ethiopia [12]. This situation, combined with an increasing burden of injuries and RTIs, makes Ethiopian families particularly vulnerable. A study from India found that households paid on average $400 and $369 for both direct and indirect medical costs for hospital care after road and non-road traffic injuries, respectively [9,14]. However, there is limited evidence on the financial risk of trauma care after RTIs in sub-Saharan Africa and Ethiopia. Having RTI as the second cause of unintentional injury [15], evidence of the economic impact of RTI in Ethiopia is important. Such evidence is highly needed when considering universal public finance and scale-up of emergency care.

Therefore, in this paper, we aim to estimate the magnitude and intensity of the burden of OOP expenditures associated with RTI care in public and private hospitals in Addis Ababa, Ethiopia's capital city.

## Methods

We conducted a hospital-based cross-sectional observational study among RTI patients who sought trauma care in Addis Ababa, Ethiopia.

### Study design and setting

Addis Ababa is the capital city of Ethiopia, with an estimated population of about 3.5 million in 2018 [16]. Addis Ababa and surrounding towns roughly account for 59–65% of all vehicles and 75% of all RTIs in Ethiopia [17], [18], [19]. There are 12 public and 46 private hospitals in Addis Ababa, but only three public and four private hospitals have a trauma care unit.

We surveyed three public hospitals (ALERT hospital, AaBET hospital, and Tikur Anbessa hospital) and one private hospital (Yordanos orthopedics hospital) in Addis Ababa.. These hospitals were selected based on their high caseload of trauma care and their geographic proximity to "black spots" (i.e., areas with frequent RTIs) [20].

All victims of RTIs seeking care as either outpatient or inpatient care at the surveyed hospitals were eligible to participate. We excluded patients on their first 24 h of care to avoid interviewing patients in time of distress for ethical reasons.

A previous study conducted in public hospitals in Addis Ababa found that about 37% of emergency visits for RTIs were severe injuries [21]. The arrangement of third-party insurance covers only 2000 ETB ($70), which is not sufficient to cover major injuries requiring further investigation, such as computerized tomography (CT) scan or advanced emergency care.

We determined a sample size of 478 patients by assuming 30% CHE among the wealthiest quintile (Q5), a 20%-point difference with the poorest quintile (Q1) and a 5% non-response rate, using the following formula building on methods from a previously published study [21]:

$n\;={\left(Z_{\alpha /2\;}+Z_{\beta }\right)}^{2}*\;\frac{p_{1}\left(1-p_{1}\right)+p_{2}\left(1-p_{2}\right)}{{\left(p_{1}-p_{2}\right)}^{2}}$, where Z_α/2_ is the critical value of the normal distribution at α/2 (i.e., for a confidence level of 95%, α is 0.05, and the critical value is 1.96), Z_β_ is the critical value of the normal distribution at β (e.g., for a power of 80%, β is 0.2, and the critical value is 0.84), and *p*_1_ and *p*_2_ are the expected sample proportions of the two groups.

We purposely selected three public hospitals based on the specialty of care they provide (predominantly trauma or referral center for trauma care) and one private hospital based on geographic proximity to the black spots mapped by road traffic authority. At the hospital level, we used the size of the patient burden of the hospitals from their 2010 Ethiopian fiscal year annual report (2017/18) to determine the sample proportion allocated to each hospital. All eligible individuals were sequentially recruited from outpatient follow-up clinics and inpatient wards until the target sample in each facility was fulfilled.

### Data collection

Data collection was conducted from December 1, 2018, to February 30, 2019. We used a structured questionnaire adapted from Tolla et al. [22]. The questionnaire was developed in English and translated to Amharic, Ethiopia's official language. The Amharic version of the questionnaire was used for face-to-face interviews with patients or their caretakers led by trained nurses (*n* = 3) and one emergency care professional to conduct the data collection after pilot testing in one public hospital in Addis Ababa. The principal investigator performed random on-site visits during interviews, checked random hospital records (*n* = 20), and made random phone calls (*n* = 20) to patients for data validation and quality assurance. We also assigned head nurses in each hospital's trauma unit to oversee the data collection activities alongside the supervisor (the principal investigator). Administered questionnaires were collected at the end of the day from the enumerators to prevent possible data manipulation.

We used exit interviews at the outpatient pharmacy or procedure room for outpatient data gathering. We gathered any expenses related to outpatient trauma care for the current and previous visits during the interview. We also asked for any costs in the prior inpatient stays for all outpatient care-seeking participants. We administered an inpatient questionnaire at the last inpatient day or discharge, in consultation with the responsible nurse in the ward for release plans. As for outpatient, we gathered information on the past expenses of outpatient care for all inpatient care-seeking participants.

Additionally, we gathered data on sociodemographic characteristics, household expenditures, household living conditions, number of outpatient visits in the past year, and financial sources to cover the cost of RTI for each survey participant. We defined household income as the average income of economically active household members from formal employment or self-employment (after tax), including any cash transfer or gift to the household. We calculated annual household expenditures from household expenses for food items, utilities, education, housing, clothing, and health care. Costs were collected in the form of either monthly average or annual expenditures, based on the household's expenditure items, and were translated into annual expenses if collected per month.

Both household expenditures and OOP expenses were collected in Ethiopian Birr (ETB) and converted into 2018 USD using the average exchange rate for the year 2018 (1 USD = ETB 28.574) [23].

### Out-of-pocket (OOP) expenditures

We projected OOP expenses from outpatient visits and inpatient admissions for which we had OOP expenditures and the total number of outpatient visits and inpatient admissions. For both inpatient stays and outpatient visits, the total OOP expenditures were the sum of OOP expenditures of direct medical costs (e.g. costs of medication, imaging and laboratory, physician consultation fees, hospital stays) and direct non-medical costs (e.g. transportation costs, attendant (caretaker) expenses, and food expenses).

### Measuring cases of catastrophic health expenditures (CHE)

We counted CHE cases at a threshold of 10% of annual household consumption and 40% of household non-food consumption. Based on the 10% threshold, a given household would experience CHE when annual OOP payments on RTI exceed 10% of annual household expenditures. To account for the fact that a large part of households’ income is spent on food items, especially in low-income settings, we also used household expenditures after food and basic necessity items were deducted (capacity to pay) [24]. In such cases, a CHE case would occur when OOP payments exceed 40% of household non-food consumption.

### Measuring incidence and intensity of CHE

We expressed the incidence of CHE as a headcount (*H*), which is the proportion of households that incurred catastrophic payments, as shown in the formula below [24]. Define an indicator $E_{i}$, which equals 1 if $\frac{T_{i}}{X_{i}}>Z$, and 0 otherwise ($T_{i}$ is OOP health expenditures, $X_{i}$ is total household expenditures and Z is a given threshold), and we count:$\;H=\frac{1}{N}\sum_{i=1}^{N}E_{i}$, where *N* is the sample size.

The CHE headcount H however fails to capture the amount by which a household can exceed the given threshold. The catastrophic payment overshoot (*O*) can capture the average degree by which a payment (as a proportion of total expenditures) exceeds the threshold Z [24]. Define the household overshoot as $O_{i}=E_{i}\left((\frac{T_{i}}{X_{i}})-Z\right)$, then the overshoot is the average given by $O=\frac{1}{N}\sum_{i=1}^{N}O_{i}$ .

### Impoverishment

We analyzed the degree of impoverishment due to OOP expenditures associated with trauma care using the household as a unit of measure with adjusting the expenses for the household composition. A household is impoverished by OOP health spending when its consumption expenditures are above the poverty line yet fall below it after deducting OOP health spending from them: this only applies to those households above the poverty line [24]. To estimate the extent of impoverishment due to OOP health spending, we used the international poverty line of $1.90 (PPP) per person per day. We categorized households along wealth quintiles using annual per capita total household consumption expenditures. We ranked the households in increasing order (from poorest to richest, where the first 20% and last 20% of households constituted the first and fifth quintiles, respectively) [24].

### Factors affecting CHE and OOP

Multivariate logistic regressions of the possible factors affecting CHE and OOP were conducted with variables including residence, income level, age of patient, diagnosis, occupation, type of health facility visited and whether or not there was hospitalization.

We also tested the significance of variations of OOP across wealth quintiles, by facility type (private vs. public), and by residence.

## Results

### Sociodemographic and economic characteristics of participants

The study recruited 478 individuals, of whom five did not agree to participate (three from public hospitals and two from the private hospital). We excluded 21 participants from the analysis because of incomplete responses. We included 452 participants (95% response rate) in the final analysis: 79 (17%) from the private hospital, and 373 (88%) from public hospitals. Participants were recruited from outpatient (*n* = 232) and inpatient units (*n* = 220), while 15% (*n* = 67) of the participants received outpatient care only and 48% (*n* = 217) received inpatient care only.

**Fig. 1:**
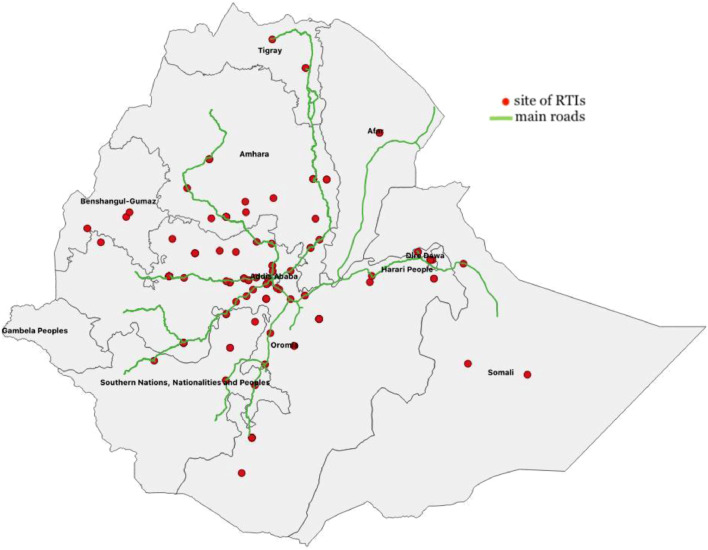
The sites of the road traffic accidents approximately located to the nearest towns.

Most study participants were men (70%) and the majority (71%) of the participants were in the age group 15–39 years. Nearly 60% of the participants came from rural or semi-urban regions of the country, and the others were residents of Addis Ababa (Fig. 1). The mean household size of participants was 4.5 (Table 1). The median and mean annual total household expenditures were $2520 and $2050, respectively, of which 46% accounted for annual food expenditures. Extremity injuries were the most common injury, accounting for 57% of the cases, followed by multiple injuries (17%) and head injuries (11%) (Table 1).

**Table 1:** Sociodemographic characteristics, types of injury, and numbers of visits of participants.

Sex	Number (%)
Male	318 (70%)
Female	134 (30%)
Age (years)	
0–14	29 (6%)
15–24	103 (23%)
25–39	216 (48%)
40–59	80 (18%)
> 60	24 (5%)
Educational level	
No formal education	56 (12%)
Primary school	163 (36%)
High school	136 (30%)
Certificate level education	42 (9%)
Degree level education +	55 (12%)
Marital status	
Married	227 (50%)
Never married	210 (47%)
Widow	9 (2%)
Divorced	6 (1%)
Occupation	
Private/Self employee	201(44%)
Student	84 (19%)
Government employee	46(10%)
Farmer	43 (9%)
Unemployed	39 (9%)
Home stay mum	35 (8%)
Retired	4 (1%)
Residence	
Outside of Addis Ababa	268 (59%)
Addis Ababa	184 (41%)
Body site of injury	
Lower limb	194 (43%)
Multiple	76 (17%)
Upper limb	63 (14%)
Head	52 (11%)
Pelvic	30 (6%)
Spinal	11 (3%)
Other	26 (5%)
Number of patients by visit type	Number
Outpatient only visit	217
Inpatient only visit	67
Inpatient/outpatient mixed visit	168
Public only hospital visit	391
Private only hospital visit	34
Public/private mixed visit	27
Household size	
Mean (SD)	4.5 (2.1)
Median	4
Annual household expenditures (USD)	
Mean (SD)	2520 (1896)
Median	2050
Annual non-food household expenditures (USD)	
Mean (SD)	1368 (1630)
Median	956

### OOP expenditures and CHE cases

**Fig. 2:**
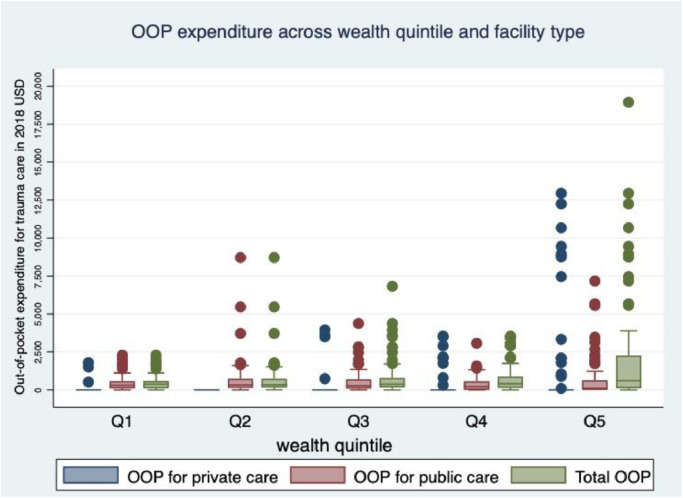
Out-of-pocket (OOP) expenditures (2018, USD) across wealth quintiles and per facility type for road traffic injury associated with trauma care in public and private hospitals in Addis Ababa, Ethiopia (2018).

The mean and median OOP expenditures for trauma care after RTI was $947 and $395, respectively. OOP expenditures for inpatient trauma care at private hospital were six times higher than at public hospital while it was threefold higher for outpatient care. The two main drivers of direct medical expenses were surgical procedures and drug expenses accounting for $102 and $91 of the average costs, respectively, and the mean attendant expenses ($96) and transport expenses ($68) were the two most important causes of the indirect medical costs (Table 2). The total transport expenses for patients and attendants from Addis Ababa was $40 in average while the total average expenses for attendants and patients coming from outside of Addis Ababa was $86. The overall average OOP expenditures increased across wealth quintiles, and the wealthiest quintile spent nearly twice as much as the poorest quintile. We saw similar patterns across wealth quintiles for inpatient and outpatient care distinctly (Fig. 2 and Table 3).

**Table 2:** Out-of-pocket expenditures (USD) on trauma care disaggregated by type of hospital visit, patient residence, and the type of expenses for road traffic injury associated with trauma care in public and private hospitals in Addis Ababa, Ethiopia (2018).

Summary	Mean (SD)	Median
Total out-of-pocket costs (*N* = 452)	947 (1843)	395
Public (*N* = 391)	625 (961)	350
Private (*N* = 34)	3430 (3716)	2100
Public & private mixed (*N* = 27)	2474 (3935)	1190
By hospital visit type		
Outpatient (*N* = 244)	444 (1317)	87
Public (*N* = 198)	282 (609)	87
Private (*N* = 19)	932 (1397)	129
Public & private mixed (*N* = 27)	1281(3264)	63
Inpatient (*N* = 435)	770 (1348)	350
Public (*N* = 351)	527 (758)	333
Private (*N* = 57)	2989 (3066)	3000
Public & private mixed (*N* = 27)	1192 (1519)	385
By residence of the patient		
Addis Ababa	881(1806)	245
Outside of Addis Ababa	992 (1869)	511
By type of expenses (*N* = 452)		
Direct medical costs		
Procedure (surgery)	102 (390)	0
Drug	91 (313)	7
Hospital stay (bed)	55 (222)	3
Laboratory & Imaging	24 (65)	1
Indirect medical costs		
Transport	68 (109)	27
Attendant expenses	96 (193)	35
Other expenses	73 (50)	0
By diagnosis at admission		
Head injury (*N* = 52)	827 (1233)	350
Upper limb injury (*N* = 63)	1104 (2621)	434
Lower limb injury (*N* = 194)	1137 (1987)	458
Spinal injury (*N* = 9)	914 (1826)	159
Pelvic fracture (*N* = 30)	1112 (1853)	604
Chest injury (*N* = 2)	2100 (1039)	2100
Abdominal injury (*N* = 1)	227	227
Other injuries (*N* = 49)	809 (1363)	470

**Table 3:** The proportion of households affected by catastrophic health expenditures (CHE), the extent of catastrophic overshoot based on different thresholds, and out-of-pocket (OOP) expenditures across wealth quintiles for road traffic injury associated with trauma care in public and private hospitals in Addis Ababa, Ethiopia (2018).

Wealth quintile	10% threshold total household consumption		25% threshold total household consumption		40% threshold (non-food consumption)		Average total household expenditure	Average OOP expenditure
	CHE %	Overshoot %	CHE %	Overshoot %	CHE %	Overshoot %		
Quintile 1	80	59	59	49	71	37	936	494
Quintile 2	70	31	45	23	60	13	1595	681
Quintile 3	64	23	40	16	55	5	2102	721
Quintile 4	59	17	35	10	41	3	2476	663
Quintile 5	62	33	44	26	49	18	3442	2184
All households	67	33	45	25	55	15	2107	947

The incidence and intensity of CHE at a 10% threshold, 25% threshold, and 40% threshold of non-food spending are shown in Table 3. The overall headcount shows that 67% of the households experienced CHE (10% threshold), while at a 40% threshold, the incidence was 56%. CHE would decrease across quintiles: the poorest quintile experienced 1.2 times higher CHE rates (10% threshold) and 1.5 times higher CHE rates (40% threshold) than the wealthiest quintile (Table 3). Mean overshoot would similarly decrease across quintiles (10% threshold) except at the 3rd and 4th quintile for the 40% threshold (Table 3).

### Magnitude of impoverishment

**Fig. 3:**
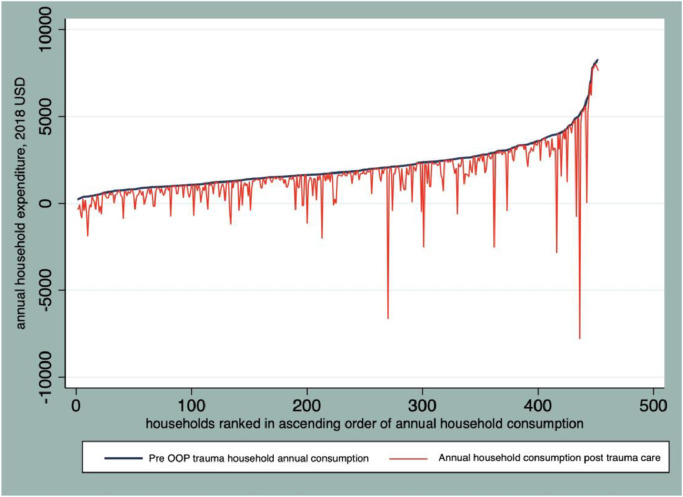
Effect of out-of-pocket (OOP) payments for road traffic injury associated with trauma care on Pen's parade of household expenditure distribution in Addis Ababa, Ethiopia (2018).

OOP medical expenses for trauma care pushed 24% of households into poverty, while 36% of households were already under the international poverty line ($1.90 per day, PPP). The poorest quintiles were the most affected. As shown on the Pen's parade graph (Fig. 3), many households are pushed below the poverty line with some remarkable spikes in each quintile.

### Coping mechanisms

Households used various coping mechanisms to overcome OOP expenditures. Household income (62% of the time) and household savings (48% of the time) were used to fund OOP expenditures. Support from friends and family was also a significant financial source for 36% of the households (accounting for $740 on average). Only 7% of the households had partial or full insurance, and another 7% obtained support from the vehicle owner or driver causing the accident. Five percent of households sold assets, whilst 7% of households borrowed money to cover their expenses.

### Factors affecting CHE and OOP expenditures

Total OOP expenses for trauma care varied by patient residence. The residents of Addis Ababa had mean OOP expenditures of $818 (median of $245), while residents from outside Addis Ababa had mean OOP expenditures of $992 (median of $ 510) (Table 1). In the multivariate analysis, four variables were associated with trauma care-related CHE: hospitalization (aOR: 10.8; 95% CI: 5.4–24.8), being in the most deprived quintile (aOR: 2.2; 1–4.9), residence outside of Addis Ababa (aOR: 2.95; 1.8–4.8) and care-seeking at private facilities (aOR: 6.5; 2.6–15.8). However, there is no statistically significant difference in CHE with the size of the patient's household, gender, age, and educational level. A one-way ANOVA analysis for degree of difference with OOP expenditures as response variable and place of first admission (government vs. private) showed a significant difference between private and government facilities;- F(1398) = 76.12; *P* < 0.001).

## Discussion

OOP expenditures and CHE cases for trauma care constitute a high proportion of household income in Ethiopia. To our knowledge, this is the first attempt to estimate the financial risk associated with trauma care after RTI in Ethiopia. Despite highly subsided care (by the Ethiopian government), the very high levels of CHE seem unique in comparison with results from similar settings (e.g., India [25]) and CHE levels associated with other health services in Ethiopia [22,32,33]. We can attribute the high CHE related to trauma care for RTI in Ethiopia to two main factors: high OOP expenditures at the point of care with limited prepayment schemes and poor access to trauma care with long waiting time [26,27].

The limited number of trauma units that are concentrated in Addis Ababa makes it more challenging to access safe and effective trauma care, especially for the poor who are more exposed to financial hardship. In our study, 60% of the participants had to travel 50–600 km to visit Addis Ababa facilities to seek trauma care. Limited access to care has raised the financial risk in our study, a finding that is similar to what was reported by Hailemichael and colleagues for mental health care in Ethiopia, where access to care was also limited to the capital city [29]. Aside from patient residence, CHE was significantly higher for patients receiving inpatient care (vs. outpatient care) and private care (vs. public care).

The occurrence of both CHE and impoverishment was more prominent in the poorest quintiles (1.5 times higher in the lowest vs. highest quintile). However, there seems to be an inverse relationship between OOP expenditures and CHE occurrence. Average OOP expenditures were nearly four times higher in the richest quintile (vs. the poorest quintile), which could be due to the greater private care-seeking behavior among the richest (21%) vs. the poorest (3%) quintile.

In line with the national plan of expansion of surgical care in Ethiopia through the saving life through surgery (SaLTs) initiative [30], universal public finance of trauma care requires due attention by policymakers in Ethiopia. Even if these services are prioritized in the country's essential health services, addressing the alarmingly increasing burden of RTI, and its financial burden requires implementing affordable and cost-effective surgical interventions.

Similar to most LMICs, Ethiopia has a weak emergency preparedness for trauma care and injury [29]. In the absence of robust financial systems to address urgent care for injuries on the road, preventive strategies are more important than ever. Several well-known and effective interventions have been proven to be of good value for money to prevent road traffic accidents [31]. Nonetheless, in Ethiopia, there is limited contextual information on the effectiveness and cost-effectiveness of such interventions. Further studies on the effectiveness of intersectoral interventions to prevent RTIs and availing trauma care with minimal financial risk are required.

Nevertheless, this study's findings have to be interpreted with caution. It is a cross-sectional hospital-based study where we tried to capture the costs incurred by patients and households for the period before the survey. Expenses incurring in the future could not be captured by this method. We also did not account for the productivity loss of the patients and their caregivers in this study. And the estimation of CHE is unable to capture the non-use and underutilization of services due to financial barriers. This could result in a potential underestimation of CHE, especially among poorer households. Lastly, recall bias pertaining to health care and consumption expenditures could affect our findings.

## Conclusions

The extent of financial risk due to RTI and seeking trauma care in Addis Ababa, Ethiopia, is alarmingly high. Despite highly subsidized care, limited coverage of trauma care and lack of financial safety nets or insurance mechanisms have a significant role in exposing Ethiopian households to significant amounts of OOP expenditures. Even though the essential health services package (EHSP) of Ethiopia [28] has prioritized interventions for treating injuries and preventing emergencies, challenges with access to trauma care need further policy attention. Intersectoral RTI prevention policies and the careful inclusion of cost-effective interventions into the community-based health insurance programs now being scaled up in Ethiopia could play a pivotal role in eliminating trauma-induced impoverishments.

## Funding

Bill & Melinda Gates Foundation through the Disease Control Priorities-Ethiopia (DCP-E) project (INV-010174) to the University of Bergen and Harvard T.H. Chan School of Public Health.

## CRediT authorship contribution statement

**Hailu Tamiru Dhufera:** Data curation, Visualization, Investigation, Conceptualization, Formal analysis, Writing – original draft. **Abdulrahman Jbaily:** Conceptualization, Formal analysis, Writing – review & editing. **Stéphane Verguet:** Data curation, Visualization, Conceptualization, Formal analysis, Writing – review & editing. **Mieraf Taddesse Tolla:** Conceptualization, Formal analysis, Writing – review & editing. **Kjell Arne Johansson:** Data curation, Visualization, Conceptualization, Formal analysis, Writing – review & editing. **Solomon Tessema Memirie:** Data curation, Visualization, Conceptualization, Formal analysis, Writing – review & editing.

## Declaration of Competing Interest

All the authors have no competing interest to declare.

## References

[1] Murray C.J., Frenk J. (2000). A framework for assessing the performance of health systems. Bull World Health Organ.

[2] Roberts M., Hsiao W.C., Berman P., Reich M. (2008).

[3] Universal Health Coverage [Internet]. World Health Organization. World Health Organization; [cited 2020 Jun 20]. Available from: https://www.who.int/health-topics/universal-health-coverage#tab=tab_1. (Accessed [24 May 2020])

[4] Wagstaff A., Flores G., Hsu J., Smitz M.F., Chepynoga K., Buisman L.R. (2018). Progress on catastrophic health spending in 133 countries: a retrospective observational study. Lancet Global Health.

[5] Wagstaff A., Flores G., Smitz M.-.F., Hsu J., Chepynoga K., Eozenou P. (2018). Progress on impoverishing health spending in 122 countries: a retrospective observational study. Lancet Global Health.

[6] Haakenstad A., Coates M., Marx A., Bukhman G., Verguet S. (2019). Disaggregating catastrophic health expenditure by disease area: cross-country estimates based on the world health surveys. BMC Med.

[7] Verguet S., Memirie S.T., Norheim O.F. (2016). Assessing the burden of medical impoverishment by cause: a systematic breakdown by disease in Ethiopia. BMC Med.

[8] Wesson H.K., Boikhutso N., Bachani A.M., Hofman K.J., Hyder A.A. (2014). The cost of injury and trauma care in low-and middle-income countries: a review of economic evidence. Health Policy Plan.

[9] Prinja S., Jagnoor J., Chauhan A.S., Aggarwal S., Ivers R. (2015). Estimation of the economic burden of injury in North India: a prospective cohort study. Lancet.

[10] Institute for Health Metrics and Evaluation (IHME). GBD Compare. Seattle, WA: IHME, University of Washington, 2015. Available from http://vizhub.healthdata.org/gbd-compare. (Accessed [24 May 2020])

[11] World Health Organization (2018). https://www.who.int/publications/i/item/9789241565684.

[12] Abegaz T., Gebremedhin S. (2019). Magnitude of road traffic accident-related injuries and fatalities in Ethiopia. PLoS One.

[13] Deme D. (2019). Road traffic accident in Ethiopia from 2007/08–2017/18. Am Int J Sci Eng Res.

[14] Federal Democratic Republic of Ethiopia Ministry of Health. August 2017. Ethiopian health accounts household health service utilization and expenditure survey 2015/2016, Addis Ababa, Ethiopia. [online] Repository.iifphc.org. Available at: <http://repository.iifphc.org/handle/123456789/408> [22 June 2020 ].

[15] Fenta T.M. (2014). Demands for urban public transportation in Addis Ababa. J Intell Transp Urban Plan.

[16] Mulugeta H., Tefera Y., Abegaz T., Thygerson S.M. (2020). Unintentional injuries and sociodemographic factors among households in Ethiopia. J Environ Public Health.

[17] Abegaz T., Berhane Y., Worku A., Assrat A., Assefa A. (2014). Road traffic deaths and injuries are under-reported in Ethiopia: a capture-recapture method. PLoS One.

[18] Tulu G.S., Washington S., Haque M.M., King M.J. (2015). Investigation of pedestrian crashes on two-way two-lane rural roads in Ethiopia. Accid Anal Prev.

[19] Behak New business Ethiopia. Ethiopia imports 135,457 vehicles in a year-News August 3, 2019 [cited on 2020 June 2]. Available from: https://newbusinessethiopia.com/trade/ethiopia-imports-135-457-vehicles-in-a-year/

[20] Addis Ababa Road Traffic Authority, Road safety annual report of Addis Ababa 2017/18; Addis Ababa 2018.

[21] Baru A., Azazh A., Beza L. (2019). Injury severity levels and associated factors among road traffic collision victims referred to emergency departments of selected public hospitals in Addis Ababa, Ethiopia: the study based on the Haddon matrix. BMC Emerg Med.

[22] Tolla M.T., Norheim O.F., Verguet S., Bekele A., Amenu K., Abdisa S.G. (2017). Out-of-pocket expenditures for prevention and treatment of cardiovascular disease in general and specialised cardiac hospitals in Addis Ababa, Ethiopia: a cross-sectional cohort study. BMJ Global Health.

[23] National Bank of Ethiopia. Inter-bank daily foreign exchange rate in (USD)-archive 2018 [cited 2019 Nov 16]. Available from: https://nbebank.com/transaction-exchange-rates-for-major-currencies-against-birr/

[24] O'Donnell O., van Doorslaer E., Wagstaff A., Lindelow M. (2008).

[25] Prinja S., Jagnoor J., Sharma D., Aggarwal S., Katoch S., Lakshmi P.V.M. (2019). Out-of-pocket expenditure and catastrophic health expenditure for hospitalization due to injuries in public sector hospitals in North India. PLoS One.

[26] Endalamaw A., Birhanu Y., Alebel A., Demsie A., Habtewold T.D. (2019). The burden of road traffic injury among trauma patients in Ethiopia: a systematic review and meta-analysis. Afr J Emerg Med.

[27] Aenderl I., Gashaw T., Siebeck M., Mutschler W. (2014). Head injury-a neglected public health problem: a four-month prospective study at Jimma University specialized hospital, Ethiopia. Ethiop J Health Sci.

[28] Federal Ministry of Health of Ethiopia (2019).

[29] Hailemichael Y., Hailemariam D., Tirfessa K., Docrat S., Alem A., Medhin G. (2019). Catastrophic out-of-pocket payments for households of people with severe mental disorder: a comparative study in rural Ethiopia. Int J Mental Health Syst.

[30] Federal Ministry of Health of Ethiopia (2016).

[31] Chisholm D., Naci H., Hyder A.A., Tran N.T., Peden M. (2012). Cost-effectiveness of strategies to combat road traffic injuries in sub-Saharan Africa and South East Asia: a mathematical modeling study. BMJ.

[32] Assebe L.F., Negussie E.K., Jbaily A., Tolla M.T., Kjell A.J. (2020). Financial burden of HIV and TB among patients in Ethiopia: a cross-sectional survey. BMJ Open.

[33] Memirie S.T., Metaferia Z.S., Norheim O.F., Levin C.E., Varguet S., Kjell A.J. (2017). Household expenditures on pneumonia and diarrhea treatment in Ethiopia: a facility-based study. BMJ Global Health.

